# Effectiveness of Video-Observed Therapy in Tuberculosis Management: A Systematic Review

**DOI:** 10.7759/cureus.71610

**Published:** 2024-10-16

**Authors:** Kartik K Sundaram, Rafdzah Ahmad Zaki, Divya Shankar, Victor Hoe, Nur Ar Rabiah Ahmad, Wong Chee Kuan, Aziezah Binti Norul Anhar

**Affiliations:** 1 Department of Social and Preventive Medicine, Faculty of Medicine, Universiti Malaya, Kuala Lumpur, MYS; 2 Department of Social and Preventive Medicine, Center for Epidemiology and Evidence-Based Practice, Faculty of Medicine, Universiti Malaya, Kuala Lumpur, MYS; 3 Department of Respiratory Medicine, Faculty of Medicine, Universiti Malaya, Kuala Lumpur, MYS; 4 Department of Public Health, Faculty of Medicine, Universiti Malaya, Kuala Lumpur, MYS

**Keywords:** compliance, telemedicine, treatment adherence, tuberculosis, video observed therapy

## Abstract

This systematic review aimed to assess the association between video-observed therapy (VOT) and treatment adherence among TB patients and the benefits and limitations of this treatment modality. The systematic review used the Preferred Reporting Items for Systematic Reviews and Meta-Analyses (PRISMA) flow guideline. Multiple databases including Cochrane, Lilacs, PubMed, Scopus, Lancet, Google Scholar, Science Direct, Directory of Open Access Journal (DOAJ), and BMC were employed to identify relevant articles published between 2012 and 2024. All data were extracted using a standardized data extraction form and both narrative and quantitative approaches were used to present the review outcomes and available evidence. Twenty-nine articles were included in the final analysis, with most using a prospective cohort (n = 10) research design. Treatment adherence rates were relatively higher in TB patients managed using VOT relative to those subjected to direct-observed therapies (DOTs). Likewise, using the VOT approach in most interventional studies lacking a control group depicted higher treatment adherence rates post-intervention. Although asynchronous VOT was used in most studies compared to the synchronous approach, the treatment adherence level was not significantly different between the two methods of VOT delivery. The predominant benefits of VOT include time-saving, cost-effectiveness, flexibility, and fewer self-reported side effects, whereas the main limitation was the privacy of patients’ data and information. Video-directly observed therapy (VDOT) is a promising approach for TB treatment with the capacity to improve adherence to medication regimes and reduce the cost of treatment, stigmatization, and burden on healthcare providers.

## Introduction and background

Tuberculosis (TB) is a significant public health concern each year. The World Health Organization (WHO) estimates that there are approximately 1.8 million lives lost worldwide to TB in 2020 alone (WHO, 2020). It was also estimated that there were 9.9 million people newly diagnosed with TB in 2020, but the actual reported numbers were only 7.1 million. The number of deaths due to TB surged to 1.8 million in 2020 from 1.4 million in 2019 [1]. 

By the year 2035, the WHO aims to attain a world free of TB via the “End TB Strategy” [2]. Direct observed therapy (DOT) has been the most widely and traditional approach to heighten TB treatment completion and adherence rates, as well as supporting better health outcomes for TB patients. The approach entails daily supervision of patients’ treatment intake, which is achieved either by using the facility-based or community-based DOT. Individuals managed using the former are required to visit the healthcare facility to take their medication for six months while being supervised by a healthcare worker [3]. Meanwhile, the supervisors for community-based DOT encompass community health workers, peer groups, and family relatives of the patient [4,5]. Nevertheless, these in-person methods are time and resource-intensive for healthcare workers and patients and the quality of care usually differs across various modes of delivering DOT. Prior reports suggest that DOT is perceived as disempowering and stigmatizing to individuals under TB treatment [6,7]. 

Poor adherence to traditional DOT is a silent menace with about one in 10 TB patients failing to complete their treatment course [8]. Logistical issues, costs, and distance to healthcare facilities are the few other factors identified for poor adherence globally. There are many factors leading to non-adherence to traditional DOTS among TB patients. A systemic review reported that the main factors associated with TB treatment non-adherence are lack of transportation cost, lack of social support, distance to clinic, and poor communication between patients and healthcare workers [9]. Numerous studies have also documented the consequences of incomplete adherence, such as a higher risk of treatment failure, onset of acquired drug resistance, and persistent infection [10,11]. As a result, programmatic strategies involving digital technologies have been proposed to improve treatment adherence [12,13].

Recent advances in digital communications technology remain a strong candidate for improved treatment adherence among TB patients globally [14]. This potential is well-recognized by the WHO Digital Health for the end TB strategy, advocating for evidence-based approaches to underpin the scale-up of new technologies. Video DOT (VDOT) is an approach whereby adherence is monitored as patients transmit digital images and videos of their ingestion of medication to a centralized location to be reviewed by supervisors. VDOT is executed either by using the synchronous method, involving real-time review of transmitted images by healthcare workers, or asynchronous in which videos are recorded, uploaded, and reviewed at a later time. Hence, the latter approach is more flexible for patients and healthcare providers [15,16].

The widespread use of technologies such as smartphones and increasing access to the Internet has created diverse platforms for the usage of VODT [16]. TB patients can take their medication in their comfort zones via video call or sending a recorded video asynchronously, rather than presenting themselves at a healthcare facility [17]. Several empirical studies have reported the effects of VDOT on treatment adherence, as well as compared between VDOT and in-person modes [18,19]. A few reviews have attempted to summarize the findings from studies reporting the use of VDOT or DOT; however, both management methods are yet to be systematically compared. Thus, evidence-based data are lacking to ascertain the effective approach for better treatment outcomes in TB patients. Although VDOT has been demonstrated to be feasible and acceptable to patients and healthcare workers in high-income settings [6,20,21], there are factors still limiting its application in diverse regions and communities globally [17]. A robust comparison of these two types of management approaches is pertinent to identify their benefits and weaknesses, as well as assist clinicians and relevant healthcare bodies in selecting the most feasible and optimal approach under different settings. Bridging these knowledge gaps requires a systematic analysis of original articles reporting the use of VDOT and DOT for the management of TB patients and the treatment outcomes. The primary objectives of this systematic review are to assess the effectiveness of VDOT and DOT for the management of TB patients based on the treatment adherence, compliance, and seroconversion rates, as well as the benefits and limitations of both treatment methods.

## Review

Methods

This systematic review was developed according to the guidelines in the Preferred Reporting Items for Systematic Reviews and Meta-analyses (PRISMA) statement with little modification. The PRISMA statement is a widely used and effective tool for conducting a comprehensive review given its provision for a concise definition of research questions, identifying eligibility criteria, and assessing relevant and accessible studies pertinent to a research topic [22]. Specifically, we used the PRIMA statement designed for synthesizing results without meta-analysis (SiWM). The main steps in developing this review comprised formulation of the research question, literature search process and eligibility criteria, study selection and screening, data extraction and manipulation, and quality appraisal or risk of bias assessment.

Formulation of the Research Question and Definition of Terms

This article aims to provide the available evidence on the effectiveness of VDOT in enhancing treatment adherence among TB patients. The other objective is to identify the limitations and benefits of this mode of treatment to aid in the development of policies for the betterment of the management of TB worldwide. In order to synthesize the research questions and strategy for the literature search process, the PICO instrument was utilized. PICO is a well-established tool for identifying the core components in a review of intervention studies, whereby P = population of interest, I = intervention, C = comparison group, and O = outcomes.

The population of interest included all patients above the age of 13 who are undergoing active TB treatment in any setting. This included patients with pulmonary, extrapulmonary, smear-positive or -negative, and drug-susceptible and -resistant TB as well as patients with latent TB. The intervention comprised VOT for the aforementioned TB patients. The comparison group entailed TB patients receiving usual care or other treatment modalities for TB, while the primary outcome measure was medication adherence.

Therefore, the research questions developed for this systematic review were as follows. 

1. What are the common outcome measures employed in assessing the effectiveness of VDOT among TB patients? 

2. Which available treatment approach (i.e., in-person/DOT vs VDOT) is the most effective for higher treatment adherence rates among TB patients?

3. What are the documented benefits and limitations of using VDOT for the management of TB patients? 

The following are the definitions we used to describe our primary outcome measures in this review. Adherence is the completeness by which a patient follows a treatment. A patient is considered to have adhered to treatment if they have completed ≥80% of the treatment [17]. Therefore, adherence rate is defined as the number of patients undergoing the treatment who have completed ≥80% of treatment [17,20]. On the other hand, the compliance rate is the percentage of completed treatment doses in comparison to the total number of prescribed doses [20]. Seroconversion is defined as a TB patient with bacteriologically confirmed TB at the beginning of treatment who was smear or culture-negative in the last month of treatment and on at least one previous occasion for two consecutive months or more [19].

Literature Search Process 

The search of our literature was limited to articles published between 2010 and 2024. We searched the literature in multiple databases including Cochrane, Lilacs, PubMed, Scopus, Lancet, Google Scholar, Science Direct, Directory of Open Access Journal (DOAJ), and BMC. The search terms combined medical subject heading (MeSH) terms and text words including “tuberculosis,” “TB,” “video observed therapy,” “VOT,” “VDOT,” “asynchronous,” “synchronous,” “telemedicine,” “mHealth,” “adherence,” “compliance,” and “seroconversion.” We also reviewed references of relevant articles and systematic reviews and contacted experts in the field for unpublished studies. 

The Boolean operators “AND” or “OR” were used in combining the keywords in the search strings, excluding the search string for Google Scholar as only the keywords were used. The search strategy and terms are presented in Table 1.

**Table 1:** Search strategy and databases

Database	Search strings
Pubmed	(Tuberculosis OR TB) AND (VOT OR "video observed therapy" OR VDOT OR asynchronous OR synchronous OR Telemedicine OR mHealth) AND (adherence OR compliance OR seroconversion)
Cochrane Library	(Tuberculosis OR TB) AND (VOT OR "video observed therapy" OR VDOT OR asynchronous OR synchronous OR Telemedicine OR mHealth) AND (adherence OR compliance OR seroconversion)
Scopus	(Tuberculosis OR TB) AND (VOT OR "video observed therapy" OR VDOT OR asynchronous OR synchronous OR Telemedicine OR mHealth) AND (adherence OR compliance OR seroconversion)
Google Scholar	video observed therapy, tuberculosis, medication adherence, seroconversion, compliance
Science Direct	(Tuberculosis) AND ("video observed therapy" OR VDOT OR synchronous OR Telemedicine OR mHealth) AND (adherence OR compliance OR seroconversion)

Eligibility Criteria and Study Selection and Screening

The inclusion criteria entailed original research papers, written in English, and published between January 2010 and April 2024. This criterion was based on the surge in studies focusing specifically on advanced technologies for medication adherence among TB patients in the last decade, as well as to increase the likelihood of retrieving more recent information on the research topic. In terms of the study population, studies must have had a form of VOT either asynchronous or synchronous with or without a control group. All studies, even those that did not compare VOT with any other form of treatment were included. To measure outcome, adherence rates, compliance rates, and seroconversion rates were included. Studies published before the year 2010, written in languages other than English, review book chapters, and non-peer-reviewed articles were excluded. 

We included all studies in the English language and rejected those that were in foreign languages. We included all randomized controlled trials (RCTs) as well as prospective and retrospective cohort studies (CSs) however we excluded all systematic reviews, meeting notes, discussions, and presentations. In addition, all research articles and abstracts that were not relevant to the keywords and those duplicated and unpublished in peer-reviewed journals were also excluded from this study. 

The title, keywords, and abstracts of all retrieved articles were independently evaluated by the authors. Meanwhile, inaccessible articles were not considered for further assessment. Articles fulfilling the inclusion criteria were subjected to full-text screening. The Cohen Kappa was computed to assess intercoder agreement following the preliminary screening, and the estimate was greater than 0.70, indicating an acceptable level of agreement [23]. Any conflict in the screening process between the authors was resolved by discussion and expert opinions. 

Data Extraction and Manipulation

Two authors participated in the data extraction process. The first author of this manuscript specified the information to be gathered from the articles, while the second author extracted the data according to the procedure applied in previous systematic reviews [4,17]. First, each article was initially examined for its relevance in answering the research questions, experimental design, a well-defined video-based protocol for the management of TB patients, and medication adherence as either the main or one of the outcomes. 

We recorded pertinent information about the study such as authors’ names and year of publication, study design and population, treatment protocol used, and main findings. The data and information extracted from numerous research articles for this study were tabulated in a standard data abstraction sheet. The information extracted from the articles that met our inclusion criteria are as follows: title, 2) author, 3) country, 4) journal, 5) year of publication, 6) objectives, 7) study design, 8) study population, 9) study sample, 10) type of VOT used, 11) study period, 12) comparator, 13) compliance rate, 14) adherence rate, 15) seroconversion rate, 16) limitations of VOT, and 17) benefits of VOT. The limitations and benefits highlighted in each article were listed narratively with the intent to identify any patterns and classify them into broader themes. All selected articles were from search engines on databases dedicated to indexing relevant medicine journals that were published between 2012 and 2023. All the extracted data were recorded in a Microsoft Excel spreadsheet.

Quality Appraisal 

We used the Cochrane Handbook for Systematic Reviews of Interventions, specifically the Risk Of Bias In Non-randomized Studies of Interventions (ROBINS-I) tool, to assess the quality and risk of bias in the articles involving experimental designs [24]. Five domains were considered for explicit reporting comprising the sequence generation, allocation concealment, blinding, and completeness of data. For the first domain, the use of a randomized sampling method was assessed either by a random number table, tossing a coin, or computerized generated numbers. Regarding the blinding of recruited patients, studies were considered adequate if both the intervention providers and outcome assessors were blinded (i.e., minimal risk of compromised blinding) and inadequate if no blinding or partial blinding was performed. For outcome measures, trials were recorded as adequate if there were no missing data or if they stated the reasons for missing data that were unlikely to be associated with the true outcome. We also assessed if the trial was free from selective reporting in which those available study protocols and all prespecified outcome measures were judged as adequate and vice versa for those not reporting all prespecified primary outcomes. Two authors performed the quality appraisal independently using a simple form and the results were presented for each study as low, unclear, or high, reflecting a low risk of bias, uncertain risk of bias, and a high risk of bias, respectively. The assessment results were compared and any discrepancies were resolved by consulting a third reviewer. The decision to include a study with an “unclear rating” was based on consensus.

Results

Study Selection 

**Figure 1:**
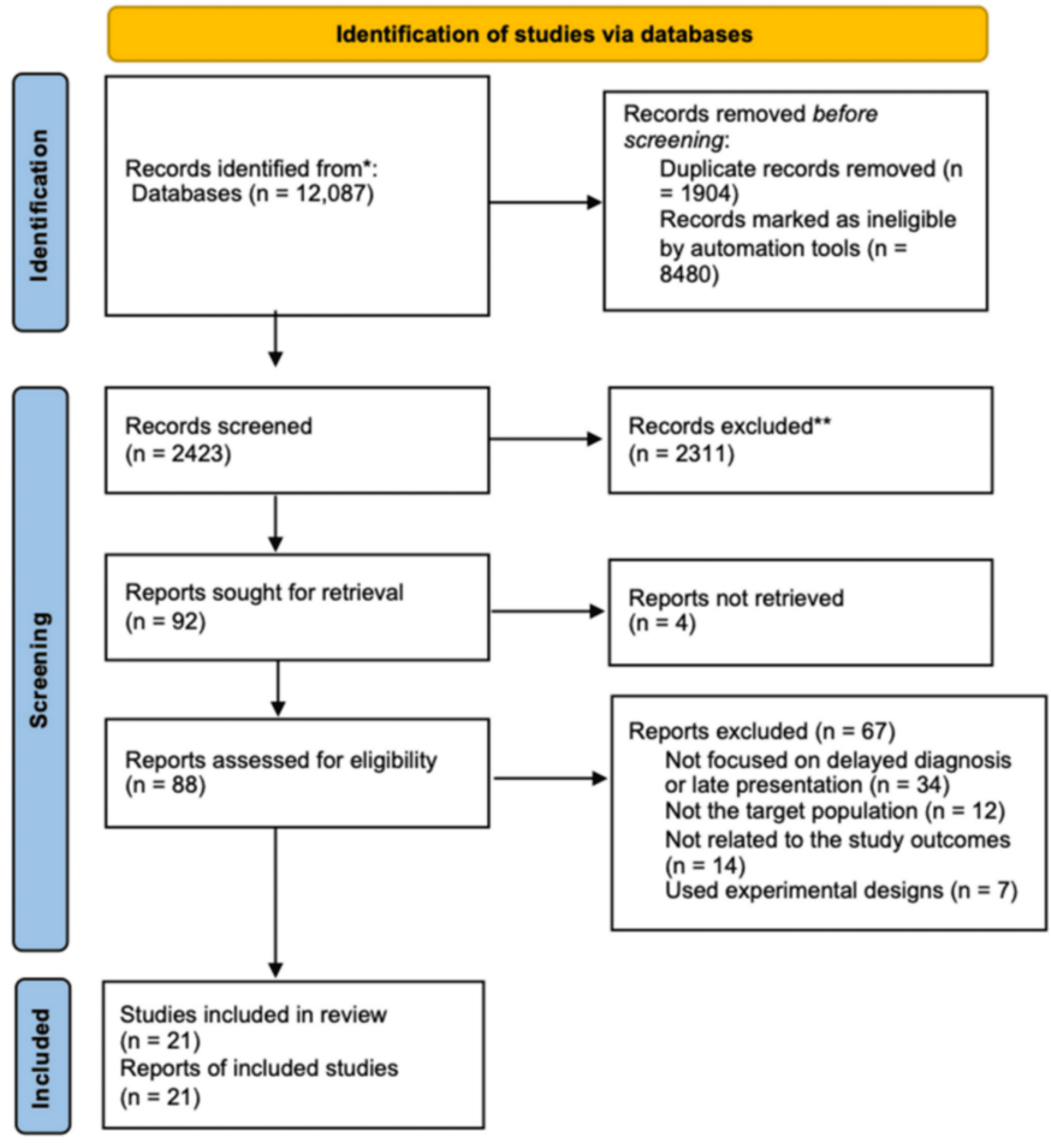
PRISMA flow diagram of the study selection process

Our search initially identified 4,803 articles, followed by the removal of duplicates and irrelevant articles (n = 2,176), which led to the synthesis of 2,627 reports for further screening. Screening of titles and abstracts yielded 103 relevant studies, which were eligible for full-text screening. The full texts of seven articles were not retrievable, hence 96 articles were subjected to full-text reading. Thereafter, 67 articles were excluded due to the following reasons: VOT was not employed for TB management and therapy (n = 32), the target population was not TB patients (n = 22), medication adherence was not the primary or secondary outcome (n = 13). Therefore, a total of 29 articles were included in the final analysis and systematic review (Figure 1).

Characteristics of the Studies 

A total of 29 eligible articles involving VOT with TB patients were included in this review as summarized in Table 2.

**Table 2:** Comparative analysis of the 29 articles included in the systematic review DOT = Direct observed therapy, CDOT = Community direct observed therapy, VDOT = Video observed therapy, VST = Video supported therapy, TB = Tuberculosis, RCT = Randomized control trials, NA = Not available, LTBI = Latent tuberculosis infection

S/n	Reference	Country	Year	Study design	Patients’ characteristics	Sample size	Duration	Comparator	Compliance rate
1	Chen et al., 2022 [5]	Taiwan	2022	Retrospective study	LTBI	445 (SVOT = 96 and CDOT = 349)	9 months	CDOTS (Compliance 61.4%, Success rate 73.12%)	66.40%
2	Garfein et al., 2018 [6]	USA	2018	Prospective Cohort	Suspected and confirmed TB patients	274	1 year	Both CDOTS and Comm DOTS (Compliance = 66%)	NA
3	Garfein et al., 2020 [7]	USA, Mexico	2020	Pragmatic single arm interventional study	All TB patients	149	1 year	None	88%
4	Story et al., 2019 [15]	England	2019	RCT	All TB patients	226	2 months	DOTS (31% Adherence, 46% Compliance)	91%
5	Guo et al., 2019 [16]	China	2019	RCT	All TB patients	405 (VOT = 203, DOTS = 202)	1 year	CDOTS (Treatment success 87.6%)	NA
6	Doltu et al., 2021 [18]	Moldova	2021	RCT	All TB patients except MDRTB with 4 months treatment left	647 (169 from previous parent study and 478 cohort)	Not mentioned	CDOTS (Adherence 19%)	NA
7	﻿Bachina et al., 2022 [19]	USA	2022	Prospective cohort	All TB patient with at least 2months of treatment left	49 (VOT = 23 and CDOTS = 26)	1.5 years (staggered into 3 phase)	Comm DOTS (54.5%)	81%
8	Mirsaeidi et al., 2015 [20]	USA	2015	Retrospective study	68 active TB patients from January 2013 to December 2014	VDOT	Not mentioned	In-person VDOT	Compliance rate not mentioned in the results
9	Rabinovich et al., 2020 [21]	Cambodia	2020	Qualitative	6 focus groups of TB patients	6 focus groups receiving SDOT	Not mentioned	No comparator	NA
10	Perry et al., 2021 [25]	USA	2021	Prospective Cohort	All TB patients	163 (VOT = 94, DOTS = 69)	11months (Each patient=aver age 27weeks)	CDOTS, SAT, Comm DOTS (Compliance 53.9%, Treatment Success 90%)	68.40%
11	Alagna et al., 2015 [26]	USA	2015	Retrospective observational cohort	Active TB Patients	11	2 years	None	97%
12	Lam et al., 2018 [27]	USA	2018	RCT	LTBI patients	352 (VOT = 50, DOTS = 302)	8 months	CDOTS (treatment completed = 64.9%)	NA
13	Kara et al., 2022 [28]	Turkey	2022	Analytic cross-sectional study	TB patients	DOT = 33, VDOT = 52	NA	Adherence rate was > 80%, higher in the VDOT than the DOT. Independent factors for stigma scores were VDOT and male gender	NA
14	Lippincott et al., 2022 [29]	USA	2022	Retrospective study	All TB patients	52	2 years	DOTS (Compliance 59%)	86%
15	Holzman et al., 2018 [30]	USA	2018	Prospective mixed methods	All TB patients	28	17 months	Both CDOTS and SAT (Compliance = 98%)	94%
16	Chuck et al., 2016 [31]	USA	2016	Prospective Observational Cohort Study	All TB patients	390 (VOT = 61, CDOT = 329)	9 months	Both CDOTS and Comm DOTS (Compliance 91%, treatment completed 97%)	95%
17	Burzynski et al., 2022 [32]	USA	2022	RCT	Suspected and confirmed TB patients	256 (mixed arms)	2 years	Both CDOTS and Comm DOTS (Compliance: 87.2%)	89.80%
18	Ravenscroft et al., 2020 [33]	Moldova	2020	RCT	All TB patients except MDRTB with 4 months treatment left	178 (CDOT = 93 and VOT = 85)	22 months	CDOTS (Adherence 19.5%, Success 90.3%)	NA
19	Rodrigues et al., 2023 [34]	India	2023	Exploratory cohort	25 adult patients with TB	25 patients	6 months	6 (64%) participants were optimally adherent but the overall adherence rate was ≥ 80%. 21 (84%) found the application easy to learn; 13 (52%) preferred vDOT over DOT. Married subjects had higher odds of adhering daily to anti-TB treatment.	NA
20	Rajahlati et al., 2022 [35]	Finland	2022	Quasi-experimental	30 patients (18 for Table VST and 12 for App VST)	TableVST vs AppVST	NA	Adherence level was > 80% in 27/30 cases. Treatment adherence was slightly greater in TabletVST relative to (98%) app VST (mean 87%).	NA
21	Nagaraj et al., 2019 [36]	India	2019	Quasi-experimental	21 patients in the intervention group and 79 from the comparison group	Intervention = 21, control = 79	NA	DOTs (90.5% vs 84.8%) at the intensive phase, 85% vs 71.4% at end of continuation phase	NA
22	Guo et al., 2020 [37]	China	2020	Unclear (treatment and retrospective control group)	TB patients	DOT group = 158, VOT = 235	NA	Retrospective control group	NA
23	Casalme et al., 2022 [38]	Philippines	2022	﻿Observational implementation study	MDR-TB patients	110	6 months	None	96%
24	Sekandi et al., 2010 [39]	Uganda	2020	Prospective Cohort	All TB patients	50	3 months	No comparator	85%
25	﻿ Wade et al., 2012 [40]	Australia	2012	﻿Retrospective cohort design	All TB patients	﻿128 patients with active TB (58 in intervention group, 70 in control group)	8 years	DOTS	NA
26	Herawati et al., 2021 [41]	Indonesia	2021	Quasi-experimental	TB patients	62 respondents in the intervention group and 60 in the control group	One-month	No intervention group	Not mentioned
27	Al Daajani et al., 2023 [42]	Saudi Arabia	2023	Prospective non-randomized interventional study.	20 TB patients	All patients in one intervention group	NA	No comparator	Not mentioned
28	Olano-Soler et al., 2019 [43]	﻿Puerto Rico	2019	Not mentioned	TB and LTBI patients	17 patients (11 active TB + 6 LTBI)	4-6 months (4months for LTBI and 6 months for PTB)	None	87%
29	﻿Anh et al., 2017 [44]	Vietnam	2017	Prospective Cohort	All TB patient with at least 2 months of treatment left	78	62 days	No comparator	88%

The majority of the studies were conducted in the USA (n = 12) [6,7,19,20,25-27,29-32], two studies each in India [34,36], and China [16,37], one study each in Mexico [6], Taiwan [5], Moldova [33], England [12], the Philippines [38], Uganda [39], Australia [40], Indonesia [41], Cambodia [21], Turkey [28], Finland [36], Saudi Arabia [42], and Puerto Rico [43]. 

In terms of methodologies, most articles were prospective cohort/longitudinal (n = 9) and retrospective (n = 6) observational study designs compared to RCTs (n = 6), quasi-experimental/single arm experiment (n = 6), and qualitative design (n = 1), whereas one study was categorized as unclear. Regarding study duration, most studies used a long-term follow-up period ranging from nine months to two years (n = 19), except for one study that is scheduled to last for eight years [40]. A few studies included a short follow-up period ranging from two months to six months (n = 8), whereas the duration in one study was unclear [21].

Assessment of Study Outcomes 

Treatment adherence and compliance rates: Either treatment adherence or compliance rates or both were reported in all the studies included in this review. A total of 17 articles entailed a comparator group, meaning comparisons between VOT and other treatment modalities such as in-person, community-DOTs. The remaining 12 studies had no comparison group. In terms of treatment adherence rate, comparisons between CDOT and VOT were performed in seven studies [15,16,18,33,36,40,41]. Overall, the treatment adherence rate was significantly higher (P < 0.05) in the VOT groups at 75.1% [33], 90% [18], 71% [15], and 85% [36] compared to the estimates in the comparison groups.

For studies comparing various treatment modalities or different time points, a significantly higher (P < 0.05) treatment adherence rate was reported in groups exposed to VOT (88%) compared to those subjected to DOTS (69%) [40]. One study revealed adherence rates of 93% and 99.5% during the two-time assessment points in their study involving VDOT [42].

Treatment adherence rates decreased from 90.5% and 84.8% in the intervention and control (VOT and DOTs) groups during the intensive phase to 85% and 71.4% during the continuation phase of their intervention, although the difference was not statistically significant (P > 0.05) [36]. In contrast, the treatment adherence rate was significantly higher (P < 0.05) in the treatment (95%) compared to the control (58%) following an educational intervention using Javanese Language videos among TB patients [41]. In terms of treatment discontinuations, a reflection of failure to adhere to treatment modalities, the DOT group recorded a significantly higher number of treatment discontinuations relative to the VOT group [16]. For studies without a comparison group, relatively lower treatment adherence rates of 50.70% and 65.5% were reported in two studies [27,43] while two other articles reported a high treatment adherence rate ranging from 93% to 100% (100%) [18,44-47].

Compliance rates were reported in 10 studies comparing VOT and other treatment modalities and five studies lacking a comparison group. The compliance rate among TB patients subjected to VOT was significantly higher than the rate in those under DOT [12,19,29]. Meanwhile, the compliance rates were similar in the VOT and other treatment groups in other studies [3,25,30-32,34]. Overall, a high compliance rate ranging from 88% to 97% was reported in studies without a comparator group [7,26,29,38,44].

Seroconversion rate: Ten studies reported the seroconversion rates among TB patients participating in the VOT. The highest seroconversion rate was 100% [26], followed by studies reporting rates between 91% and 97% [3,25,31,33,34,37], and those slightly below 90% [27,38]. One study compared the seroconversion rate between TB patients monitored using DOT and VOT, with the latter group recording a higher rate at 48% relative to DOT at 33% [40]. 

VOT Delivery Methods

VOT as mentioned can be classified into two types, synchronous (live video observation) and asynchronous (recorded). Out of 29 studies, 10 studies involved synchronous VOT, 18 used asynchronous and one study used both methods. For the asynchronous VOT, the platforms used included smartphones, tablets or computers but these specific methods were mentioned only in a few studies. The treatment adherence recorded in studies using asynchronous VOT ranged from 88.4% to 98%. Whereas the platform used for synchronous VOT was rarely mentioned in the reviewed studies, the treatment adherence was equally high, ranging from 64% to 80%.

Limitations and Benefits of VOT 

Based on thematic analysis, seven categories of limitations were identified from the usage of VOT for TB treatment. As shown in Supplementary Table 1, the predominant issue was the privacy of patients’ data and information, connectivity, and less-informed users. Other limitations include problems with technology, phone-related issues, and lack of human touch or not being human-friendly. Most of these limitations were raised by TB patients, whereas healthcare workers highlighted problems relating to the timing of synchronized VOT, video and audio quality, limited time to verify the video, ineffectiveness in monitoring specific treatment-related data and constituting additional burden (Table 3).

**Table 3:** Limitations of video observed therapy for the management of patients with tuberculosis

Issue	No of studies	References
Connectivity	7	[4,26,30,31,33,37,38]
Privacy	8	[7,15,18,26,34,35,38,39]
Less informed patients/users	7	[4,18,21,30,31,44]
Lack of technology	4	[21,27,32,39]
Phone related issue	3	[4,27,31]
Lack of human touch	1	[34]
Others (Timing of synchronised VOT was not feasible, Poor video and audio quality Interpersonal issue, Limited time to verify the video, Ineffectiveness in monitoring specific treatment-related data, additional burden)	7	[12,25,27,30,31,34,38]

The additional benefits of VOT for TB treatment and management were categorized into 10 themes as shown in Supplementary Table 2. The predominant benefits include time-saving, cost-saving, convenience/flexibility/usability, followed by satisfaction with VOT, less self-reported side effects, stigma reduction, and logistic/less resource demanding. Other benefits such as wider coverage, the propensity to learn, and a patient-centered approach were mentioned in a few studies (Table 4).

**Table 4:** Benefits of using video-observed therapy for tuberculosis management

Items/themes	Sub-themes/elements	References
Satisfaction with VOT and Comparisons between VOT and DOT	Higher levels of satisfaction with VOT compared to DOT	[20,33,42]
Time-saving	Time spent in completing the therapy both from the patient's and healthcare workers’ perspectives	[4,16,31,33,34,38]
Cost	Transportation and usage costs	[3,4,16,19,26,27,30,31,33,38,40]
Coverage	Both rural and urban areas	[6,7]
Side effect	Fewer reports of side effects in VOT relative to DOT, prompt detection of side effects	[33,39,40]
General convenience/flexibility/usability	Ease of using and implementing the therapy with existing healthcare facilities	[3,25-27,29,34,37,39]
Logistic/less resources	Both human and non-human resources are required to perform VOT	[19,26,31,35]
Stigma reduction		[19,28,30,35,38]
Propensity to learn	Willingness to learn despite not being technologically savvy	[33]
Patient-centered	Focusing on the patients and designed to address the challenges faced during therapy	[26,38]

Quality assessment of the studies

We deployed two instruments, STrengthening the Reporting of OBservational studies in Epidemiology (STROBE) Checklist and ROBINS-I tool, to assess the quality of observational and experimental studies in this review. Most of the observational studies depicted a moderate level of bias (n = 9), while three articles each demonstrated a low risk and high risk of bias, respectively. The main reasons for the high risk of bias were failure to present sample size calculation, statistical tests applied for controlling confounding factors, and sample selection methods (Table 5).

**Table 5:** Quality assessment for observation studies using the STROBE checklist tool STROBE - STrengthening the Reporting of OBservational studies in Epidemiology

Criteria	Chen et al., 2022 [5]	Garfein et al., 2018 [6]	﻿Bachina et al., 2022 [19]	Mirsaeidi et al., 2015 [20]	Rabinovich et al., 2020 [21]	Perry,et al., 2021 [25]	Alagna et al., 2015 [26]	Kara et al., 2022 [28]	Lippincott et al., 2022 [29]	Holzman et al., 2018 [30]	Chuck et al., 2016 [31]	Rodrigues et al., 2023 [34]	Casalme et al., 2022 [38]	Sekandi et al., 2010 [39]	Wade et al., 2012 [40]	Anh et al., 2017 [44]
Abstract and introduction																
Title or the abstract contained the study design and commonly used terms	Yes	Yes	Yes	Yes	Yes	Yes	Yes	Yes	Yes	Yes	Yes	Yes	Yes	Yes	Yes	Yes
The abstract is informative and summarizes the research accordingly	Yes	Yes	Yes	Yes	Yes	Yes	Yes	Yes	Yes	Yes	Yes	Yes	Yes	Yes	Yes	Yes
Scientific background and rationale for the research are well-explained	Yes	Yes	Yes	Yes	Yes	Yes	Yes	Yes	Yes	Yes	Yes	Yes	Yes	Yes	Yes	Yes
Research objectives and hypotheses are clearly stated	No	No	Yes	No	No	No	Yes	No	Yes	No	No	Yes	No	No	No	Yes
Methods																
Vital elements in the study design are presented early in the article	Yes	Yes	Yes	Yes	Yes	Yes	Yes	No	Yes	Yes	Yes	Yes	Yes	Yes	Yes	Yes
Clear description of the research setting, location, relevant dates, and recruitment period. The exposure, data collection and follow-up periods are also stated	Yes	Yes	Yes	Yes	Yes	Yes	Yes	Yes	Yes	Yes	Yes	Yes	Yes	Yes	Yes	Yes
Present the eligibility criteria and sampling methods	Yes	Yes	Yes	Yes	Yes	Yes	Yes	Yes	Yes	Yes	Yes	Yes	Yes	Yes	Yes	Yes
All outcomes, treatments or exposures, independent variables, effect modifiers and potential confounders are clearly defined	Yes	Yes	No	Yes	Yes	Yes	No	No	No	Yes	Yes	No	Yes	Yes	Yes	No
Each variable is supported by the data sources and detailed information on the assessment or measurement methods	Yes	Yes	Yes	Yes	Yes	Yes	Yes	Yes	Yes	Yes	Yes	Yes	Yes	Yes	Yes	Yes
Efforts to mitigate potential sources of bias are described	Yes	Yes	Yes	Yes	Yes	Yes	Yes	Yes	Yes	Yes	Yes	Yes	Yes	Yes	Yes	Yes
Sample size calculation is explained	No	Yes	No	No	No	Yes	No	No	No	No	Yes	No	No	No	Yes	No
All statistical methods are described, including how potential confounders, missing data and interactions were addressed	Yes	No	No	Yes	Yes	No	No	No	No	Yes	No	No	Yes	Yes	No	No
Results																
Characteristics of participants	Yes	Yes	Yes	Yes	Yes	Yes	Yes	Yes	Yes	Yes	Yes	Yes	Yes	Yes	Yes	Yes
Outcome data are provided over time, and each exposure category or summary measures	Yes	Yes	Yes	Yes	Yes	Yes	Yes	Yes	Yes	Yes	Yes	Yes	Yes	Yes	Yes	Yes
Unadjusted and confounder-adjusted estimates and precision levels (95% CI) are presented. Highlight the adjusted confounders with clear reasons	NA	NA	Yes	NA	NA	NA	Yes	NA	Yes	NA	NA	Yes	NA	NA	NA	Yes
Discussion																
Important findings are summarised	Yes	Yes	Yes	Yes	Yes	Yes	Yes	Yes	Yes	Yes	Yes	Yes	Yes	Yes	Yes	Yes
Research limitations are discussed and considering imprecision and sources of potential bias	Yes	Yes	Yes	Yes	Yes	Yes	Yes	Yes	Yes	Yes	Yes	No	Yes	Yes	Yes	Yes
The extrapolation of the results is discussed (generalisability and external validity)	Yes	No	Yes	Yes	Yes	No	No	No	Yes	Yes	No	Yes	Yes	Yes	No	Yes

Four of the six quasi-experimental studies depicted a moderate level of bias, two were considered low quality while four of the RCTs demonstrated moderate to low quality, mainly due to bias in controlling for baseline and time-varying confounders, participants' selection, adjustment for missing data and outcome measurement (Table 6).

**Table 6:** Risk of bias assessment of the experimental studies using the ROBINS-I tool Y = Yes, N = No, NA = Not available ROBINS-I - Risk Of Bias In Non-randomized Studies of Interventions

Criteria and sub-questions	Story et al., 2019 [15]	Guo et al., 2019 [16]	Garfein et al., 2020 [7]	Doltu et al., 2021 [18]	Lam et al., 2018 [27]	Burzynski et al., 2022 [32]	Ravenscroft et al., 2020 [33]	Rajahlati et al., 2022 [35]	Nagaraj et al., 2019 [36]	Guo et al., 2020 [37]	Herawati et al., 2021 [41]	Al Daajani et al., 2023 [42]
Bias due to confounding												
Is there potential for confounding of the effect of intervention in this study?	N	N	N	N	N	N	N	N	N	N	N	N
Was the analysis based on splitting participants’ follow up time according to intervention received?	Y	Y	Y	Y	Y	N	Y	Y	Y	Y	Y	Y
Were intervention discontinuations or switches likely to be related to factors that are prognostic for the outcome?	N	NA	NA	N	N	N	N	NA	N	N	NA	NA
Baseline confounding and time-varying confounding												
Did the authors use an appropriate analysis method that controlled for all the important confounding domains?	Y	Y	`N	Y	Y	Y	NA	NA	Y	Y	Y	Y
Did the authors control for any post-intervention variables that could have been affected by the intervention?	N	N	N	N	N	N	Y	Y	Y	Y	Y	N
Did the authors use an appropriate analysis method that controlled for all the important confounding domains and for time-varying confounding?	Y	N	N	N	N	Y	NA	NA	Y	Y	Y	Y
Bias in selection of participants into the study												
Was selection of participants into the study (or into the analysis) based on participant characteristics observed after the start of intervention?	N	N	N	N	N	N	NA	N	N	N	N	N
Do start of follow-up and start of intervention coincide for most participants?	Y	N	Y	Y	Y	N	Y	Y	Y	Y	Y	Y
Bias in classification of interventions												
Were intervention groups clearly defined?	Y	N	Y	Y	Y	N	Y	Y	Y	Y	Y	Y
Was the information used to define intervention groups recorded at the start of the intervention?	N	N	N	N	N	Y	Y	N	Y	Y	Y	Y
Could classification of intervention status have been affected by knowledge of the outcome or risk of the outcome?	Y	N	N	N	N	N	N	N	N	N	N	N
Bias due to deviations from intended interventions												
Were there deviations from the intended intervention beyond what would be expected in usual practice?	N	Y	N	N	N	N	N	N	N	N	N	N
Effect of starting and adhering to intervention												
Were important co-interventions balanced across intervention groups?	Y	N	Y	Y	Y	Y	Y	Y	Y	Y	Y	Y
Was the intervention implemented successfully for most participants?	Y	Y	Y	Y	Y	Y	Y	Y	Y	Y	Y	Y
Did study participants adhere to the assigned intervention regimen?	Y	Y	NA	NA	NA	Y	Y	NA	Y	Y	NA	Y
Bias due to missing data												
Were outcome data available for all, or nearly all, participants?	Y	Y	Y	Y	Y	Y	Y	Y	Y	Y	Y	Y
Were participants excluded due to missing data on intervention status?	Y	Y	N	N	N	Y	N	N	Y	Y	N	Y
Were participants excluded due to missing data on other variables needed for the analysis?	Y	Y	N	N	N	Y	N	N	Y	Y	N	Y
Bias in measurement of outcomes												
Could the outcome measure have been influenced by knowledge of the intervention received?	N	N	N	N	N	N	N	N	N	N	N	N
Were outcome assessors aware of the intervention received by study participants?	N	N	Y	Y	Y	Y	N	Y	NA	Y	Y	Y
Were the methods of outcome assessment comparable across intervention groups?	Y	Y	Y	Y	Y	Y	Y	Y	Y	Y	Y	Y
Were any systematic errors in measurement of the outcome related to intervention received?	N	N	N	N	N	N	N	N	N	N	N	N
Is the reported effect estimate likely to be selected, on the basis of the results, from												
- multiple outcome measurements within the outcome domain?	N	N	N	N	N	N	Y	N	N	N	N	N
- multiple analyses of the intervention-outcome relationship?	N	N	N	N	N	N	N	N	N	N	N	N
- different subgroups?	N	N	N	N	N	N	Y	N	N	N	N	N

Discussion

This systematic review is among the few attempts to explore the application of VOT for the treatment and management of TB, with emphasis on its effectiveness, benefits and limitations. We identified a total of 29 studies published between 2010 and 2024 fulfilling the inclusion criteria, which entailed articles reporting either treatment adherence or compliance rates among TB patients subjected to VOT.

Most of the studies used observational designs such as prospective cohort/longitudinal (n = 10) and retrospective (n = 6) compared to experimental designs such as RCTs (n = 6) and quasi-experimental/single-arm trials (n = 5). This finding may stem from the challenges associated with conducting interventional research among TB patients, whereby the recruitment and allocation process into various treatment groups and controls may be time-consuming and logistically demanding. On the other hand, observational study designs require either snap-shot data or no manipulation of the patients, which may contribute to the higher number of studies employing the approach for VOT research.

In terms of study location, the majority of the studies were conducted in the USA and other developed and high-income countries such as the UK, Australia, Saudi Arabia, China, and Taiwan. A few studies were also carried out in middle to low-income countries including Moldova, India, Indonesia, the Philippines, Cambodia, and Uganda. While developed countries are expected to be more resourceful in conducting interventional research among TB patients relative to resource-limited countries, the diverse study locations reflect the wide distribution of the disease globally and the growing interest in using VOT as a treatment modality. In addition, the implementation process of VOT to facilitate treatment adherence in TB patients seems to be an important topic in TB-endemic countries.

This result differs from the findings reported in a previous systematic review, focusing on the interventions to increase the adherence level of MDR-TB patients [45]. The authors found that the retrieved articles were predominantly in low-middle-income countries, where TB is commonly associated with poor living conditions, poverty and low access to healthcare facilities. In contrast, the present review focuses on the use of VOT - a more advanced and digitalized approach for TB management, which may not be readily available in resource-limited countries.

VDOT is a technology-based management approach with the capacity to enhance treatment adherence in TB patients. The approach facilitates video conferencing technology, thereby allowing clinicians to observe and monitor patients remotely, which may lead to better treatment outcomes. As depicted in most of the reviewed studies, TB management requires prolonged treatment regimens and poor adherence to such treatment can culminate in persistent transmission, drug resistance, and mortality [17]. Poor treatment adherence constitutes a major barrier to TB control as it reduces the cure rate of TB patients [21]. Innovative approaches such as VOT are potential candidates to enhance the care and prevention required for more effective treatment.

We found that treatment adherence rates were higher in TB patients managed using VOT relative to those subjected to DOTs [16,36,40,42]. Likewise, the use of the VOT approach in interventional studies lacking a control group depicted higher treatment adherence rates post-intervention [6,41,43]. A similar result pattern was observed in a few studies reporting the compliance rate to VOT [15,19,29]. These levels of treatment adherence usually reflect patient satisfaction and better feasibility and acceptability of VDOT in different healthcare systems. Nevertheless, the slight differences in adherence results between the studies could be explained by variations in the sampled population and technological infrastructure.

We also explored the method of VOT delivery in the reviewed studies, particularly the usage of synchronized (i.e., real-time and live-stream video conferencing during the time of taking the medication) and asynchronized approaches. (i.e., recorded videos which are subsequently reviewed by healthcare workers). VDOT entails the use of digital technology platforms such as smartphones, computers, and tablets to verify the observation and monitoring of a TB patient, either in real-time or recorded while undergoing TB treatment [17,46].

In this review, most studies used asynchronous rather than synchronous VOT, but the treatment adherence level was not significantly different. Nevertheless, more complaints were raised among TB patients subjected to synchronous relative to synchronous VOT. This finding was mainly linked to the latter approach makes provision for videos to be uploaded by the patients at a later time when an internet network is available. Meanwhile, network connectivity plays a major role in conducting synchronous VOT. According to the WHO, synchronous VDOT should be considered as an alternative to in-person or community DOT only when technology is available to support the approach and toolkits developed by healthcare providers to guide clinicians in utilizing the approach with their patients [1,2].

Apart from the treatment adherence and compliance rates, several benefits of VDOT were identified in this review ranging from cost-effectiveness, less time-consuming, convenience, fewer reports of side effects, stigma reduction and less resource demanding. Some studies also revealed that TB patients considered VDOT to be patient-centered and facilitate wider coverage. Previous research has shown that VDOT is a patient-oriented and cost-effective approach that is applicable under diverse TB treatment settings for a specific group of patients.26,38 In studies comparing VDOT and DOT, the latter constituted a significant cost and time burden while also detracting from a patient-centered approach due to fixed-time commitments [5,6]. Medication adherence is equally affected by the distance between the patient’s location and the nearest healthcare facility, ease of access to healthcare services, and the patient’s knowledge of the significance of treatment [7,18,31]. Meanwhile, advantages of DOT such as patient interaction and reinforcing commitment to therapy can also be achieved via VDOT.

The frequency of side effects in TB patients undergoing VDOT or DOT represents an important finding and is worth discussing. A lower frequency of self-reported side effects may stem from improved treatment adherence and compliance rates, which are associated with better treatment outcomes in the long term [38,39]. While this event was consistently reported in VDOT patients compared to other treatment approaches in most studies, a few studies revealed contradicting findings (i.e., higher reports of side effects). Nevertheless, more side effects could be reported by VOT patients because more medicine was taken. The new approach, which entailed digitalised platforms, may also encourage regular reporting of side effects compared to the DOT [25]. Moreover, reporting side effects is promising given its importance to ascertain when medical attention or a change in therapy is required. This vital aspect of improving the quality of care is among the benefits of digital adherence technologies, including VOT.

The stigma needs to be taken into account when using digital technologies for TB management [19,29]. TB-associated stigma is a well-established social determinant of health with negative impacts on patients, including delays in seeking care and non-adherence to medication [35]. The present review depicted that patients perceived VDOT as more confidential and less likely to be seen by unauthorized individuals [6,7]. Despite the limited evidence, these results suggest that TB patients perceived employing VDOT as more private and confidential in terms of their TB status [40].

Despite the aforementioned benefits of VOT, we identified some limitations of using the treatment approach for TB patients, particularly in resource-limited countries. For instance, issues relating to poor knowledge of TB and the importance of treatment adherence, phone-related, video quality, and technological problems were predominantly raised in low- and middle-income countries. Meanwhile, issues associated with patients’ data privacy and security, limited time for video verification, and assessment of specific treatment-related data were mostly highlighted by TB patients and healthcare workers in developed and high-income countries. These findings depict the diverse factors that could shape the feasibility and acceptability of VOT in various countries, thereby indicating the need for country or regional-specific approaches. For instance, several studies demonstrated that VDOT is a feasible and effective method of TB treatment, with high levels of patient satisfaction and treatment adherence [33,42]. Nonetheless, the same studies found that in-person DOT was preferred over VDOT, with cultural differences in patient expectations and preferences playing pivotal roles. 

TB knowledge is strongly associated with the perception and practices of TB preventive and control measures [18,21,44]. Research has shown that a positive correlation exists between perception and medication adherence to anti-TB drugs. Thus, it is not surprising that education interventions were identified as the predominant interventions, especially in low- and middle-income countries [17]. Promoting positive behavioral change among people regarding TB and empowering communities to change is crucial for improved TB control. Recent research has also emphasized better interpersonal communication skills between healthcare workers and TB patients [38], which has been simplified via using mobile and software technology such as VOT.

The barriers in terms of technology and phone-related issues are expected in resource-limited countries since TB mainly affects patients with low socio-economic status [47]. Thus, technological solutions like VDOT may not be suitable for all types of patients, particularly aged and less-informed patients. Notwithstanding, findings from resource-limited countries suggest that smartphone-based technologies are still relevant in such settings as evidenced by the increasing prevalence of smartphone ownership in these countries [48,49]. A few studies also identified data privacy and security concerns as potential factors that could limit the uptake of VOT by some TB patients. Further research is indeed required to investigate approaches that reduce the risk of intrusiveness of monitoring technologies. Lastly, healthcare workers raised pertinent issues relating to VOT, particularly the limited time to observe recorded video. Factors, such as heavy work burden due to extensive care for patients with multiple comorbidities, may explain this finding.

This systematic review has identified the effectiveness of VDOT as a management approach and promising alternative to in-person methods of treatment. Most prospective and experimental studies focusing on the effects of VDOT on treatment adherence and compliance rates among TB patients recorded significant improvement in the outcome measures post-intervention. These positive effects were equally reflected in studies comparing VDOT and in-person approaches. Improvement in treatment adherence and compliance rates is vital in TB management by enabling better treatment outcomes and minimizing the risk of acquired drug resistance, persistent infection and mortality.

The support for VDOT presents an opportunity to implement this digitalized and advanced treatment modality in ways that suit the specific context of extant healthcare systems. Given the aim to achieve person-centered care which emphasizes the patient as an individual, the benefits of VDOT such as increased flexibility, convenience, cost-effectiveness, and stigma reduction facilitate a care plan that aligns with fulfilling and addressing the TB patient’s needs beyond their medical needs. Examples include patient autonomy towards their treatment plan by having a stronger control of their time, and finances relating to seeking care, and privacy. On a broader perspective, the VDOT process also offers specific advantages that take into account resource constraint healthcare systems while focusing on a patient-centered approach. Nonetheless, alternative approaches need to be developed for patients with unique needs to address the issue of privacy and confidentiality and ensure person-centered care in some settings.

The limitations of VDOT identified in this review are possible factors that could shape the implementation and uptake of VDOT in various countries. Though implementation of VDOT in place of traditional DOT offers several advantages, the high burden of TB among people of lower socio-economic class, aged individuals, poor knowledge of TB, and issues with technology and Internet availability need to be considered accordingly [18,44]. Therefore, the collection of baseline information and determining the readiness of the community and target groups for VDOT constitute the primary steps to be taken before attempting to implement such digitalized TB management systems. Alternative video platforms or training programs may be required to enhance patient ease of use and uptake of VDOT under such settings. This is particularly important in resource-limited communities whereby patient preferences and acceptability of other modes of TB management, including non-supervised approaches, are scarcely investigated. Educating patients, family relatives, and communities regarding TB and the treatment approaches while improving access to patient-centered care is equally recommended to improve treatment adherence in resource-limited countries.

Despite the strengths of this review in terms of scope, methodology, and synthesis of the results, some limitations need to be acknowledged. This review was designed to retrieve relevant articles within the timeframe (2012-2024), we recognize that it is limited in scope and breath, and thus, other nuances expressed by TB patients and healthcare providers regarding VDOT in other studies might not be captured.

Upon reviewing the included studies in this review, the type of TB and the total duration of treatment were not stated in several articles. Therefore, we could not elucidate whether certain types of TB are associated with different challenges when using VDOT, particularly those with longer treatment regimes. In addition, only a few studies reported the specific types of asynchronous or synchronous VDOT used, which made it difficult to understand patients’ and healthcare workers’ perspectives on these TB treatment modalities. Finally, it was challenging to assess the bias in the reviewed studies given the different study methodologies, which include RCT, quasi-experimental, and observational designs (i.e., cross-sectional, prospective longitudinal, retrospective, and qualitative).

## Conclusions

Overall, this review suggests that VDOT is a promising approach for TB treatment with the capacity to improve adherence to medication regimes and reduce the cost of treatment, stigmatization, and burden on healthcare providers. In addition, VDOT can be successfully integrated into individualized case management and patient-centered plans that culminate in high treatment adherence and success rates. Nevertheless, the feasibility and implementation of this digitized monitoring system need to be ascertained under different settings, as well as identifying the potential drawbacks and best practices for its implementation in TB management programs. Cultural and contextual factors that may influence its feasibility and acceptability are also important.
